# Observation based climatology Martian atmospheric waves perturbation Datasets

**DOI:** 10.1038/s41597-022-01909-y

**Published:** 2023-01-03

**Authors:** Jie Zhang, Qianqian Ji, Zheng Sheng, Mingyuan He, Yang He, Xinjie Zuo, Zefeng He, Zilin Qin, Gangyao Wu

**Affiliations:** 1 College of Meteorology and Oceanography, National University of Defense Technology, Changsha, China; 2 High-tech Institute, Fan Gong-ting South Street on the 12th, Qingzhou, ShanDong China

**Keywords:** Atmospheric dynamics, Atmospheric dynamics

## Abstract

The Martian atmospheric waves perturbation Datasets (MAWPD) version 2.0 is the first observation-based climatology dataset of Martian atmospheric waves. It contains climatology-gridded temperature, gravity waves, and tides spanning the whole Martian year. MAWPD uses the Data INterpolating Empirical Orthogonal Functions method (DINEOF) reconstruction method for data assimilation with the observational data from the Mars Global Surveyor (MGS), Mars Reconnaissance Orbiter (MRO), Mars Atmosphere and Volatile EvolutioN (MAVEN), Mars Pathfinder (MP), Mars Phoenix Lander (MPL), Mars Exploration Rover (MER) and Mars Express (MEX) temperature retrievals. The dataset includes gridded fields of temperature (Level 1 data) as well as the physical quantities of GWs (Level 2 data, amplitude, and potential energies), SPWs and tides (Level 2 data, amplitude, and phase). The MAWPD, based entirely on multiple reliable observations, provides climatological background atmospheric information of temperature and wave disturbances on Mars. The dataset is not only useful for observation-based scientific studies concerning Martian atmospheric waves, e.g., circulation, dust storms, and wave excitation mechanism, but also for cross-validating with model-based datasets or model results.

## Background & Summary

The importance of gravity waves (GWs), stationary planetary waves (SPWs), and tides for the atmospheres of Mars are now universally recognized^1–8^. In some cases, these waves act together to modify the atmosphere^9^, e.g. GWs and zonally modulated thermal tides jointly contributed to the significant wave activity over the Martian tropics^10^. In other cases, they interact with each other, e.g., nonmigrating tides are believed to be partly excited by the interaction between the migrating tides and SPWs^11–13^. Consequently, a study of these waves as a whole may better explain most of the atmospheric phenomena on Mars, which needs a dataset with a comprehensive estimate of the state and temporal evolution of the atmospheric waves. However, no dataset for Martian atmospheric waves has been established so far partly due to the scattered and discontinuous observation data that is not sufficient to build the dataset, either in time or space^4,14^ and the data scarcity had caused the difficulty in wave discrimination in the past^15^. To fill in the missing data without losing the Spatiotemporal continuity information in it required for wave analysis, the Data INterpolating Empirical Orthogonal Functions method (DINEOF) was used. The DINEOF method extracts the temporal information by using the mean of data time series to get principal components of incomplete data, which served as eigenvectors of a covariance matrix^16–18^. This ensures the temporal information is not lost in the reconstruction of data without any a priori information, which is unattainable for general methods such as Optimal Interpolation (OI)^19,20^ and Spline interpolation^21,22^. It was proved that the DINEOF method has stronger anti-noise capability, requires less computational time, and is thus more robust^23–25^. In addition, this method has been applied in the reconstruction of some terrestrial oceanographic^16,17,25,26^ and ionospheric^27,28^ datasets.

**Fig. 1:**
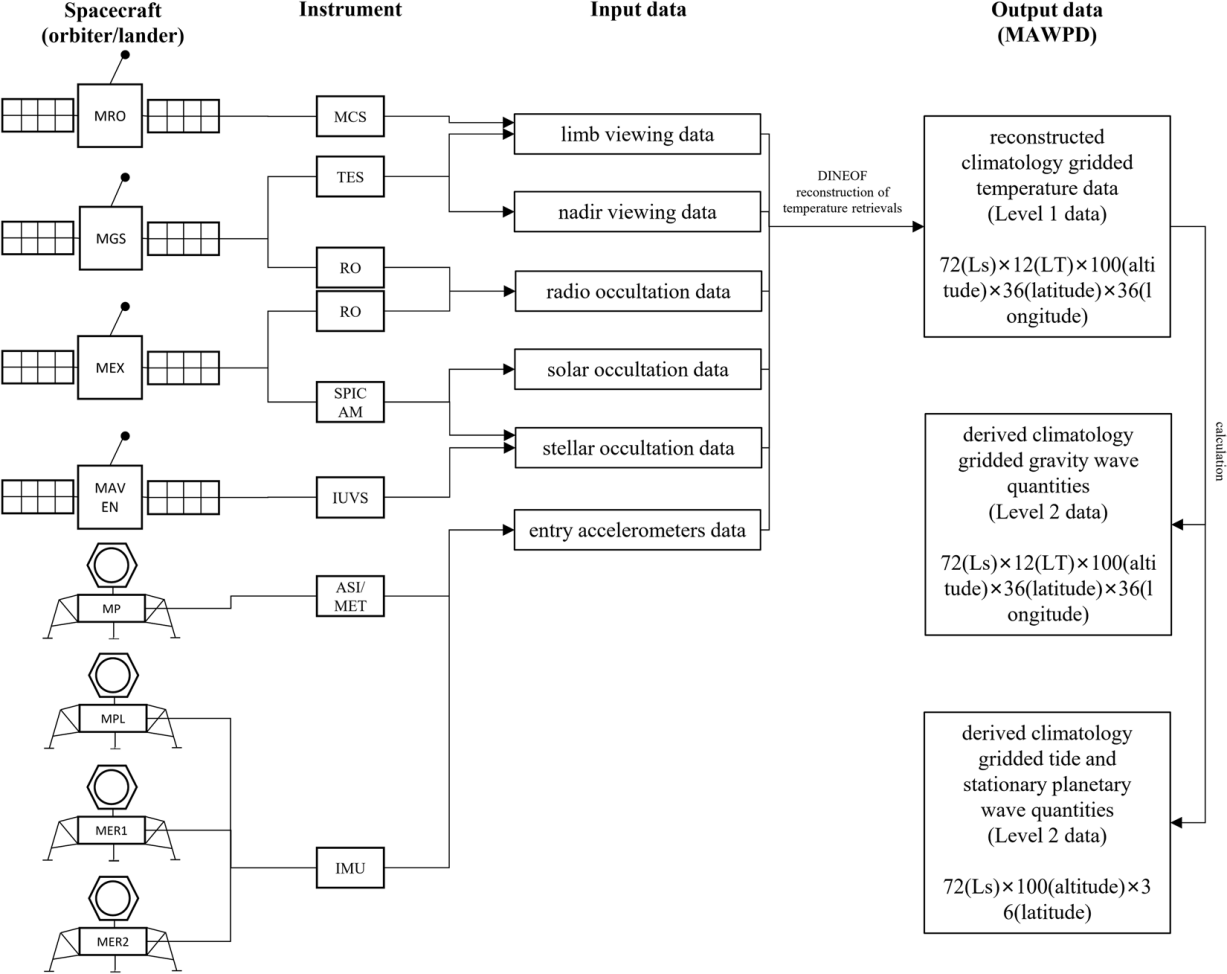
Dataset creation process. Based on the input data (third column) collected by instruments (second column) of spacecrafts (first column), the output data (final column) is reconstructed using DINEOF method. The spacecrafts used include the orbiters (MRO, MGS, MEX, and MAVEN) and the landers (MP, MPL, MER1, and MER2). They are connected to the detection instruments (MCS, TES, RO, SPICAM, IUVS, ASI/MET, and IMU) they carry by polylines. The arrows connected to each instrument point to the data type (limb viewing data, nadir viewing data, radio occultation data, solar occultation data, stellar occultation data, and entry accelerometers data) they provide. The output data (MAWPD) is the reconstructed climatology gridded temperature data (Level 1 data) and the derived gravity waves, tides, and stationary planetary waves (Level 2 data).

We established the Martian atmospheric waves perturbation Datasets V2.0^29^ (MAWPD V2.0) to address some of the aforementioned limitations noted above (Fig. 1). Based on the temperature retrievals of various spacecraft including Mars Global Surveyor (MGS)^30^, Mars Reconnaissance Orbiter (MRO)^31,32^, Mars Atmosphere and Volatile EvolutioN (MAVEN)^33,34^, Mars Pathfinder (MP)^35^, Mars Phoenix Lander (MPL)^36^, Mars Exploration Rover (MER)^37^, and Mars Express (MEX)^38–40^, the DINEOF reconstruction is conducted to gain more complete climatology gridded temperature data. Then the gained temperature (Level 1 data) is used to calculate the GWs, SPWs, and tides (Level 2 data). It aims to provide a comprehensive estimation of the climate mean state of temperature (level 1), GWs, SPWs, and tides (level 2) throughout a Martian year for scientific studies concerning Martian atmospheric waves, including atmospheric circulation, dust storms, polar warming, and the wave excitation mechanism in Mars.

It is worth mentioning that the dataset is the first Martian atmospheric dataset based only on observation. Many excellent Martian atmosphere datasets had been established on the model^41^ or the reanalysis^42–44^ data with great accuracy. But we believe that MAWPD^29^ is useful when cross-validating with model-based datasets and conducting climatology Martian atmospheric wave research.

## Methods

The MAWPD^29^ is based on the grids established by Martian atmospheric temperature retrievals from instruments on multiple orbiters and landers, while the DINEOF reconstruction method is used to fill in the missing data of grids. The reconstructed grids are then used to calculate all the waves including tides, GWs, and SPWs.

### Step1: Data acquisition and preparation of Multiple Spacecraft

To fully exploit the existing observation data, various spacecraft are used. Based on the temperature profiles of the radio occultation (RO)^45^ and Thermal Emission Spectrometer (TES)^46–49^ data of MGS^30,50,51^, the Mars Climate Sounder (MCS)’s nearly continuous limb viewing data of Mars Reconnaissance Orbiter (MRO)^31,32^, the Imaging Ultraviolet Spectrograph (IUVS)’s occultation data^52,53^ of MAVEN^33,34^, the RO^54^ and Spectroscopy for the Investigation of the Characteristics of the Atmosphere of Mars (SPICAM)^38,55^ data of MEX^56^, the entry ACC data of MP^35,57^, MPL^36,58^, MER1 and MER^37,59^ (in order of the number of profiles used), the climatology gridded temperature data is calculated.

In the dataset, the MGS/TES data including two hundred and thirty million TES profiles (both the nadir- and limb-geometry) covering from Ls = 103.1° of Mars Year (MY) 24 to Ls = 159.3° of MY 27 continuously with time interval less than the order of 10^−1^ solar longitude (Ls) and the MGS/RO data including 21087 RO profiles covering from Ls = 34.7° of Mars Year (MY) 27 to Ls = 237.3° of MY 28 discontinuously are used to retrieve temperature profiles. The nadir-geometry data have a relatively higher horizontal spatial resolution but its retrieval is limited to daytime regions and fails to cover the relatively cold polar cap, while the limb-geometry data has limited horizontal spatial resolution without geographic and time restrictions^60,61^. The MGS performed a 3.6-cm wavelength RO experiment with the signal transmitted by the spacecraft and received by a tracking station of the NASA Deep Space Network (DSN)^62^. The base of a typical RO profile is 500 m, the top is at the 10-Pa pressure level (40 km) with the sample spacing of 700 m, providing accurate measurement, especially during diametric occultation^30^.

Besides, nearly 5 million profiles from the MCS Derived Data Record (DDR) across a range of time from Ls = 111.3° of MY 28 to Ls = 38.9° of MY 36 continuously with time intervals less than the order of 10^−3^ degree of Ls are used. The observation covers the altitude range from approximately 0 to 85 km and the local time (LT) is mainly between 1–5 and 13–17 due to the special in-track and cross-track or off-track observations of MCS. The two observation methods are performed alternately to get more LT coverage, which enables the coverage of six LTs in the lower and middle latitudes and seven or more LTs in the high latitudes^4^. Spatially, MCS is designed to acquire high vertical resolution, horizontally contiguous measurements (5 km vertical and 8.6 km horizontal at the limb^32^). Its appropriate resolution and LT coverage are suitable for research of waves, having been conducted on tides^4,15,63^, GWs^64,65^, and SPWs^15,66^ in several studies. The IUVS onboard MAVEN has also detected wave perturbations at altitudes between 20 and 140 km. We use 1193 temperature profiles in the middle atmosphere during the period from Ls = 314° MY 32 to Ls = 309.6° of MY 35 with a latitude range from 70° N to 80° S. As the geometric parameters of the tangential trajectory of the occultation were changing during the whole task, the variations obtained here include these geometrical changes as well as real atmospheric variations^53^. All 1004 profiles of MEX are used, including 389 profiles of MEX/RO covering from Ls = 34.7° of MY 27 to Ls = 237.3° of MY 28 discontinuously and 615 of MEX/SPICAM covering from Ls = 332.8° of MY 26 to Ls = 37.6° of MY 28 discontinuously. The MEX/RO data provides the temperature profiles below the tropopause (~40 km), while the SPICAM data within 20–95.5 km is used here. For most of the missions to date, the periapsis of MEX has been in the dusk hemisphere, resulting in MEX sampling of the bow shock primarily in the dawn hemisphere of Mars^67^. But the MEX orbit provides excellent (nearly symmetric) coverage in both the northern and southern hemispheres of Mars, covering both hemispheres of Mars^56^.

The dataset also used the entry accelerometers data obtained by the weather station named Atmospheric Structure Instrument/Meteorology Package (ASI/MET) onboard the MP (within Ls = 142.7° of MY 23) and the Inertial Measurement Units (IMU) onboard the MPL (within Ls = 88° of MY 29), MER1 (within Ls = 339° of MY 26), and MER2 (within Ls = 327° of MY 26)^68^. Despite the limited number of profiles, the high resolution of entry accelerometers data is beneficial to sustain the information of small-scale fluctuations like GWs. The trajectory reconstruction procedures of the entry accelerometers data of MP had enhanced its accuracy and can derive atmospheric temperature relatively consistent with the PDS archive without any other aerodynamic information^69^. The atmospheric density, pressure, and temperature of MPL^70^ and MER^71^ were calculated from the same reconstructed trajectory with MP.

### Step2: Filtering out global dust storm activity

The entire premise of the dataset as a climatological average of all years requires that abnormal years must be excluded to ensure the universality of the results. Mars experiences regional dust storms, whose exact timing varies somewhat through the year, and interannually variable global dust storms. These storms affect temperatures by up to 60 K locally as well as gravity wave activity, as demonstrated by papers from the Mars Climate Sounder team or recently by researchers^72,73^.

In our present work to present interannual variability in a merged climatology, results are mainly affected by 25 and 28 years of global dust storms, and to a lesser degree by regional dust storms that occur in autumn and winter every year. To improve the issue, the second half (Ls 180–360) of the MY 25 and 28 are deleted to filter out global dust storm activity.

Regional dust storms are preserved due to its relatively slight impacts compared to the global dust storm and that it can happen throughout the year, so it is difficult to remove and does not make much sense to results after removal. In addition, wave activities during regional dust storms are meaningful to study so the removal of the global dust storms could also make sure that regional dust storm effects were not confounding.

#### Step3: Super observation and weighting of data from Multiple Spacecraft

The main issue here is to solve that how much certain datasets were weighted relative to others. For instance, the entry profiles of the MER spacecraft were used, and their ability to sample gravity wave activity was noted. However, these profiles are one per spacecraft. They would be a drop in the bucket on a single day compared to the > 1000 profiles per day provided by Mars Climate Sounder. If they are not somehow weighted, there would be no point in including them all. And indeed, the same concern would apply to radio occultation data. A thermal mapping instrument like TES or MCS makes two to three orders of magnitude more profiles than an occulting instrument. There is almost no point in using occulting instrument data if there is no weighting algorithm being applied; only the thermal mappers will matter. Therefore, the dataset needs weighting of data from Multiple Spacecraft.

This dataset is divided into two types of data (lander data and orbiter data) according to the spacecraft type, and weighted according to the data type. Lander’s entry accelerometers data are given the highest weight due to their accuracy, i.e., when there are both lander and orbiter data at the same point (at specific Ls, Lt, Altitude, Latitude, Longitude), only the lander at that point is considered and not the orbiter. When there is only orbiter data at the same point, the results of the orbiter are considered. Data of the same type have the same weight here, that is, when there is both MCS and TES data at the same point, the two are averaged.

To accomplish the weighting of instruments according to the observation error, we create the super observations^44,74,75^ for each grid. The super observation, assumed to be at the grid centre, is the weighted average of all types of inner observation. The larger the observation error, the smaller the weight of the observation. Specifically, for each grid, if the observation errors are the same, the super observation value is the mean observation value; or the observation values with smaller (larger) error would have greater (smaller) weight when averaging. The disagreements in the temperature between datasets that result from instrumental or sampling biases are handled in the process. The mean standard deviation in each grid is used as the super observation standard deviation, which is useful in determining the general quality of data and is used as a proxy for the super observation error of grid here. The super observation standard deviation of temperature in each grid is provided in the data file ‘MAWPD_v2.0_T.nc’ for users’ reference.

The sources of the adopted observation error should be mentioned. The MCS, MGS/RO, MEX/RO, MEX/SPICAM, MPL, and MER1/2 all provide 1-sigma uncertainty along with the temperature data, while the MP provides the 3-sigma uncertainty (easily converted to 1-sigma uncertainty). However, the TES observations in the PDS did not provide estimates of the observation uncertainties but selected best quality (estimated instrument-noise uncertainty < 1%) temperature data. As the TES nadir atmospheric temperature for pressure levels greater than 0.1 mbar must be between 100 and 300 K while the temperature below (tropospheric air temperature) would barely exceed 300 K, the instrument-noise is then < 3.0 K (300 K·1%). Considering the random error, it is reasonable to assign a TES observation error of 3.0 K to the super observations, as EMARS team did^44^.

### Step4: DINEOF reconstruction and data integration

The DINEOF method is used in the MAWPD^29^ to fill the missing data. This method is based on the Empirical Orthogonal Function (EOF) to track features of the Martian atmosphere with high spatial and temporal variability. While the EOFs are obtained by a Singular Value Decomposition (SVD) representation of the data matrix X (L × M × N). The L represents the length of vertical spatial dimension, M represents the length of horizontal spatial dimension and N represents the length of temporal dimension, e.g., a pixel of such data X is the data *X*_*l,m,n*_ at *m*-th zone in the horizontal section and *l*-th altitude of X in the *n*-th time. As the DINEOF was designed to work with two-dimensional fields evolving in time, the reconstruction of the three-dimensional Martian atmosphere is done by calculation of the horizontal section of the three-dimensional atmosphere at each height on a section-by-section basis. These horizontal sections at the *l*-th altitude are recorded as X_*l*_. Consequently, the SVD of each X_*l*_ is *X*_*I*_ = *UDV*^*T*^. Let matrix *I* be the set of missing data points and $(m0,n0)\in I$ represents that there is no observation available or the observations are unreliable in *X*_*l,m0,n0*_, then the element *X*_*l,m0,n0*_ corresponding to the flagged missing data at the *l*-th altitude of m0-th zone in the n0-th time could be replaced by the result of EOF decomposition with only the first N EOFs as the following equation:

1 $${X}_{l,m0,n0}=\mathop{\sum }\limits_{i=1}^{{\rm{N}}}{\rho }_{i}{({{\bf{U}}}_{i})}_{m0}{({{\rm{V}}}_{i}^{{\rm{T}}})}_{n0},(m0,n0)\in I$$

Repeat the replacement until the DINEOF reconstructions converge, which demands the variation of EOF decomposition amount obtained in 2 consecutive SVD less than 10^−8^. Since the reconstruction of the Martian atmosphere involves the reconstruction of a four-dimensional matrix of altitude, latitude, longitude, time in high resolution actually, the Lanczos method^25,26^ was chosen to ensure the effectiveness of DINEOF application.

**Fig. 2:**
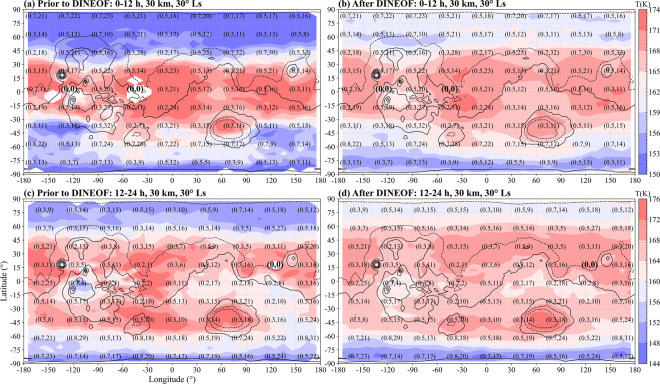
Global distributions of climate mean state of Martian atmospheric air temperature for 0–12 (**a, b**) and 12–24 (**c, d**) LT at 30 km altitude and 30° Ls before (left) and after (right) DINEOF reconstruction. Topography is shown in 3,000 m increments with the negative contours dot-dashed. The number of instruments (I) and data point (D) sampled every four grid points are used to show how heavily the grid data is constrained by data, denoted by (I,D) (see Methods).

After reconstruction, the temperature data is integrated into a matrix (Ls × Lt × Altitude × Laitutde × Longitude) for followed calculation. It is worth mentioning that all temperature data used are reconstructed from the EOFs, but results in the grid points constrained by observation data would definitely be more reliable. Here, the number of instruments (I) and data point (D) in each grid are used to show how heavily the grid data is constrained by data, denoted by (I,D) as shown in the Fig. 2. For example, a grid detected by MCS and TES (I = 2) simultaneously with each contributing two data points (D = 2I = 4) has the (I,D) of (2,4), while a grid totally reconstructed from the EOFs without being constrained by data has the (I,D) of (0,0) (see the unobserved grids of (0,0) in the Fig. 2a,c).

The more data points in the grid point, the more heavily the grid is constrained by data. But with the same number of data points, the results in the grid with more instrument types are generally more reliable as long as its instrument error is not too large. We have saved the variable I (instrument number) and D (data point number) in the ‘MAWPD_v2.0_T_I.nc’ and ‘MAWPD_v2.0_T_D.nc’, respectively.

### Step5: Gravity waves calculation

In this paper, the relative perturbation and the potential energy of gravity waves are used to characterize the intensity of gravity wave activity. To get the value of gravity wave potential energies of each profile, it is necessary to extract the perturbations caused by gravity wave from background temperature firstly, given by $T{\prime} =T-\bar{T}$ where the wave-induced perturbations *T*′ are calculated by subtracting the background temperature $\overline{T}$ from the measured instantaneous temperature *T*.

The seventh-order polynomial fit, which has been widely applied to extracting the gravity wave in the terrestrial^76–78^ and Martian atmosphere^79,80^ with credibility, is used to obtain the background temperature in the dataset at first. Then, the perturbations are filtered using a 6th-order Butterworth filter with a bandpass width of 3–15 km to remove the effects of large-scale waves including tides and planetary waves. Considering that the vertical wavelength of gravity waves on Mars reported by previous studies does not exceed 15 km^10,80–82^, we then choose a vertical bandpass filter in the range of 3–15 km. In addition, data points where the potential energy is greater than 5000 J/kg or is negative^83–85^ is deleted as outliers. The relative perturbations ($\delta T\,/\bar{T}$) of gravity waves are get by normalizing the filtered perturbations with the background temperature.

The gravity wave potential energy is used to measure the magnitude of perturbations in the dataset,

2 $${E}_{p}=\frac{1}{2}{\left(\frac{g}{N}\right)}^{2}{\left(\frac{{T}^{{\prime} }}{\bar{T}}\right)}^{2}$$

where *N* is Brunt-Väisälä frequency in Eq. (3), g is the acceleration of gravity, and *c*_*p*_ is the specific heat at constant pressure, which is usually taken as 0.844 kJ/(kg·K) for the Martian atmosphere,

3 $$N=\sqrt{\frac{g}{\bar{T}}\left(\frac{\partial \bar{T}}{\partial z}+\frac{g}{{c}_{p}}\right)}$$

Our work focuses on the extraction of low and intermediate-frequency gravity waves. The vertical wavelength scales of the gravity waves extracted from the reconstructed temperature profiles are in the range of 3–15 km due to band-pass filtering.

### Step6: SPWs and Tides calculation

The SPWs and tides are calculated by^8,15,86^:

4 $$T(\lambda ,\phi ,h,{t}_{LST})=\sum _{\sigma }\sum _{s}{T}_{\sigma ,s}(\phi ,h)\cos \left((s-\sigma )\lambda +\sigma {t}_{LST}+{\varphi }_{\sigma ,s}\right)$$

where λ, Φ, p, and t_LST_ are four dimensions of the temperature data (T), namely longitude, latitude, height, and universal time (LT at 0° longitude), respectively. While s is the zonal wavenumber and *σ* is the frequency (units of sol^−1^), thus *T*_*σ*,*s*_ and *φ*_*σ,s*_ are corresponding amplitude and phase of the wave (*σ*, *s*), the wavenumber m is calculated by $m=| \sigma -s|$. The modes of SPWs and tides were summarized well in charts that are worthy of reference in previous research^15,87,88^. The notation ‘DW + number’ or ‘DE + number’ is used to denote a westward- (W) or eastward- (E) propagating diurnal (D) tide with given zonal wavenumber. For semidiurnal oscillations, S replaces D. Thus, the diurnal westward propagating tides with the zonal wavenumber of 1 is recorded as DW1, while the semidiurnal eastward propagating tides with the zonal wavenumber 2 is recorded as SE2. The diurnal and semi-diurnal zonally symmetric (S) oscillations are denoted DS0 and SS0, respectively. The migrating tides are the westward propagating tides with equal zonal wavenumbers and the frequency (day^−1^) of the wave, e.g., the DW1 and SW2. In addition, we use ‘SPW + number’ as a substitution for the stationary planetary wave, and the number is the zonal wave number. A spectral decomposition method called FFT (fast Fourier transform) is used for each wave mode to extract the climate state wave from the Martian temperature. Due to the regularly distributed dimensions of the model results, the FFT method could obtain each wave (*σ*, *s*)^89^.

### Step7: Data storage

At last, we use MATLAB to save all variables into a single NetCDF file called ‘MAWPD_XX.nc’, and more detailed description of the variables and file are in the followed ‘Data Records’ section.

### Custom code availability

MATLAB (R2022a) and python (3.6.6) are the major applications used to process the data. The python (3.6.6) is the main tool used to read (and visualize) the dataset. The codes for pre-processing, reading the NetCDF file, plotting and calculation are stored in the GitHub (https://github.com/jiezhangMars/The-Martian-atmospheric-waves-perturbation-Datasets-MAWPD-) and zenodo^90^ (10.5281/zenodo.7395240).

## Data Records

The MAWPD^29^ has been deposited in the Dryad open-access world data centre, which is publicly available and can be downloaded at 10.5061/dryad.59zw3r2bh. The ‘MAWPD_XX.nc’ file contains three kinds of variables including ‘dimension variables’, ‘Five dimensional variables’, and ‘Three dimensional variables’ (see also the Brief Data Descriptor in the supplementary file for further information).

Specifically, dimension variables ‘Solar_Longitude’, ‘Local_Time’,’ Altitude’, ‘Latitude’, and ‘Longitude’ are five dimensions called Ls, Lt, Altitude, Latitude, Longitude, respectively.

Five dimensional variables ‘Temperature’, ‘GW’, and ‘E’ are the temperature (K), gravity wave amplitude normalized by background temperature (%), and potential energy of gravity wave (J*kg-1) in the entire covered spatio-temporal domain. Five-dimensional variable ‘Temperature’ represents the climatology atmospheric state in the entire domain completely and sustain the calculation for ‘GW’, and ‘E’. Five-dimensional variable ‘GW’, and ‘E’ represents the climatology gravity waves in the entire domain. The five-dimensional variables ‘Temperature’, ‘GW’, and ‘E’ are storage in ‘MAWPD_v2.0_T.nc’, ‘MAWPD_v2.0_GW_NA.nc’, and ‘MAWPD_v2.0_PE.nc’, respectively.

Three dimensional variables ‘amp_T_XXX’ and ‘phs_T_XXX’ are the daily and zonally mean amplitude and phase of the specific tidal wave. For example, ‘amp_T_DW1’ and ‘phs_T_DW1’ are the daily and zonally amplitude and phase of the migrating diurnal tide (DW1). Three-dimensional variables are calculated by the Five-dimensional variable ‘Temperature’. They lost two dimensions (Lt and Longitude) due to the tidal calculation described in the Eq. (4) above and represents the climatology zonal and daily mean tides in different Ls, Altitude, and latitude. They are all storaged in ‘MAWPD_v2.0_tide.nc’.

Based on the aforementioned 3.95 billion grid points of nearly 182.18 million temperature profiles after quality control obtained by spacecraft instruments (MGS/RO, MGS/TES, MRO/MCS, MAVEN/IUVS, MEX/RO, MEX/SPICAM, MP, MPL, MER1, and MER2), the high-resolution temperature grids are reconstructed with DINEOF method as Level 1 data. The grids of Level 2 data, i.e., GWs, SPWs, and tides, are calculated using the Level 1 data. The dataset resolution is 5°(latitude) × 10°(longitude) in the horizontal and 100 uniformly distributed layers in the vertical from 1 km to 100 km with per 5° Ls and 2 hr LT temporal resolution. Thus, the grid dimension is 72(Ls) × 12(LT) × 100(altitude) × 36(latitude) × 36(longitude) as shown in the table in the supplementary Brief Data Descriptor.

The Tides in the NetCDF file include the amplitude and phase of wave number 1 to 5 westward propagating migrating diurnal tide (DW1 to DW5), wave number 1 to 5 eastward propagating migrating diurnal tide (DE1 to DE5), wave number 1 to 5 westward propagating migrating semidiurnal tide (SW1 to SW5), wave number 1 to 5 eastward propagating migrating semidiurnal tide (SE1 to SE5) and diurnal zonally symmetric tide (DS0). While the SPWs products include the amplitude and phase of wave number 1 to 5 stationary planetary waves. In addition, the amplitude and phase of zonal mean temperature are calculated for reference. The GWs perturbation and potential energy representing the intensity of GWs activity are also stored as Level 2 data in the dataset.

## Technical Validation

It should be pointed out that the distribution of Martian atmospheric observation data is very uneven in space-time. The data is dense in some time and space but scarce in others (e.g., the MCS data is mainly between 1–5 and 13–17 due to its cross-track or off-track observation method). Fully capturing the signature of the available data, the DINEOF algorithm extracts the physical characteristics of the data in the space and time domains through the empirical orthogonal function, effectively reconstructing the missing physical observation data with the optimal number of modes. Consequently, the DINEOF could fill in the missing point when there is a large gap in the data with accuracy as long as the total observation rate exceeds 20% (29.68% in the dataset).

### Effects of DINEOF reconstruction

In order to obtain the climate state of the Martian atmosphere with limited coverage of observation, the DINEOF reconstruction method was used to fill the missing part and give the results a high degree of confidence. Remarkably, the distribution of Martian atmospheric observation data is very uneven in space-time. Restricted by the observation method, the data is dense in some LT and space but scarce in other LT and space (e.g., the MCS data is mainly between 1–5 and 13–17 due to its cross-track or off-track observation method). But the general interpolation algorithm has no way to fill data when there is a large gap in the data, let alone guarantee the quality of the data after the vacancies are filled. The DINEOF algorithm extracts the physical characteristics of the data in the space and time domains through the empirical orthogonal function, and can effectively reconstruct the missing physical observation data by retaining the optimal number of modes while maintaining the temporal and spatial distribution characteristics of the physical data. Consequently, the DINEOF could fill in the missing point when there is a gap in the data with great accuracy.

Data of the edge and inner part of the Tharsis Bulge^91,92^ (43°S-55°N and 60–145°W), for instance, is under severe incompleteness circumstance near the equator within 0–12 local time at 30 km altitude and 30° Ls (Fig. 2a). But after reconstruction, the data become complete and a mixture of wavenumber 2 and wavenumber 3 modes is evident in the fields prior to DINEOF (Fig. 2a) and thereafter (Fig. 2b). Such a distribution is very familiar in observations and largely reproduced in models as persisting, if shifting, through the day. In addition, the data incompleteness near the Hyblaeus Fossae (centred at 20°N and 140°E) in the southwest of Elysium Mons is also filled (Fig. 2d) without the loss of general wavenumber 2 and wavenumber 3 modes prior to DINEOF (Fig. 2c).The DIEOF method can complete the data while maintaining the temperature distribution characteristics of the middle and low latitudes. The information in the time domain is passed between different moments while filling in the missing data, which enables the reconstruction of data when there is a large missing gap at a certain momen.

### Performance of representing seasonal scale features of Martian atmospheric waves

**Fig. 3:**
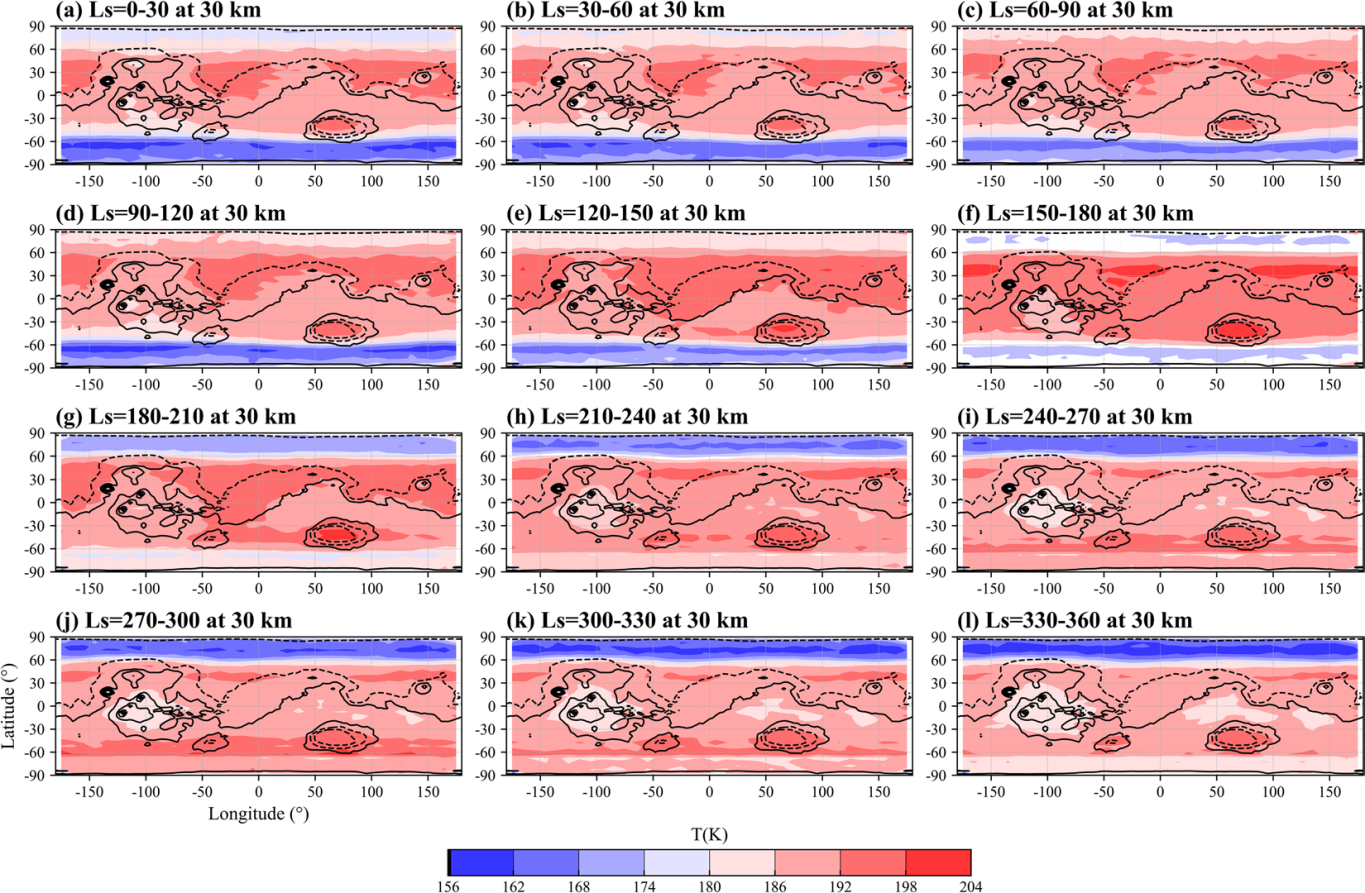
Global distributions of climate mean state of Martian atmospheric air temperature for 0–30° (**a**), 30–60° (**b**), 60–90° (**c**), 90–120° (**d**), 120–150° (**e**), 150–180° (**f**), 180–210° (**g**), 210–240° (**h**), 240–270° (**i**), 270–300° (**j**), 300–330° (**k**), 330–360° (**l**) Ls at 30 km altitude. Topography is shown in 3,000 m increments with the negative contours dot-dashed.

The climatology distributions of the reconstructed temperature (Level 1 data) represent the zonal and meridional signatures of temperature (Fig. 3). The high-temperature region in the mid and low latitude areas at 30 km, the cold area in the south (north) polar region before (after) the autumnal equinox, the relatively cold Tharsis Rise (~−100° longitude), the relatively warm Amazonis Planitia (~−150° longitude), Chryse Planitia (~−25° longitude), Hellas basin (~75° longitude) and Utopia Planitia (~120° longitude) as well as the seasonal changes in temperature due to solar radiation, circulation, and topography, can be seen in the results.

**Fig. 4:**
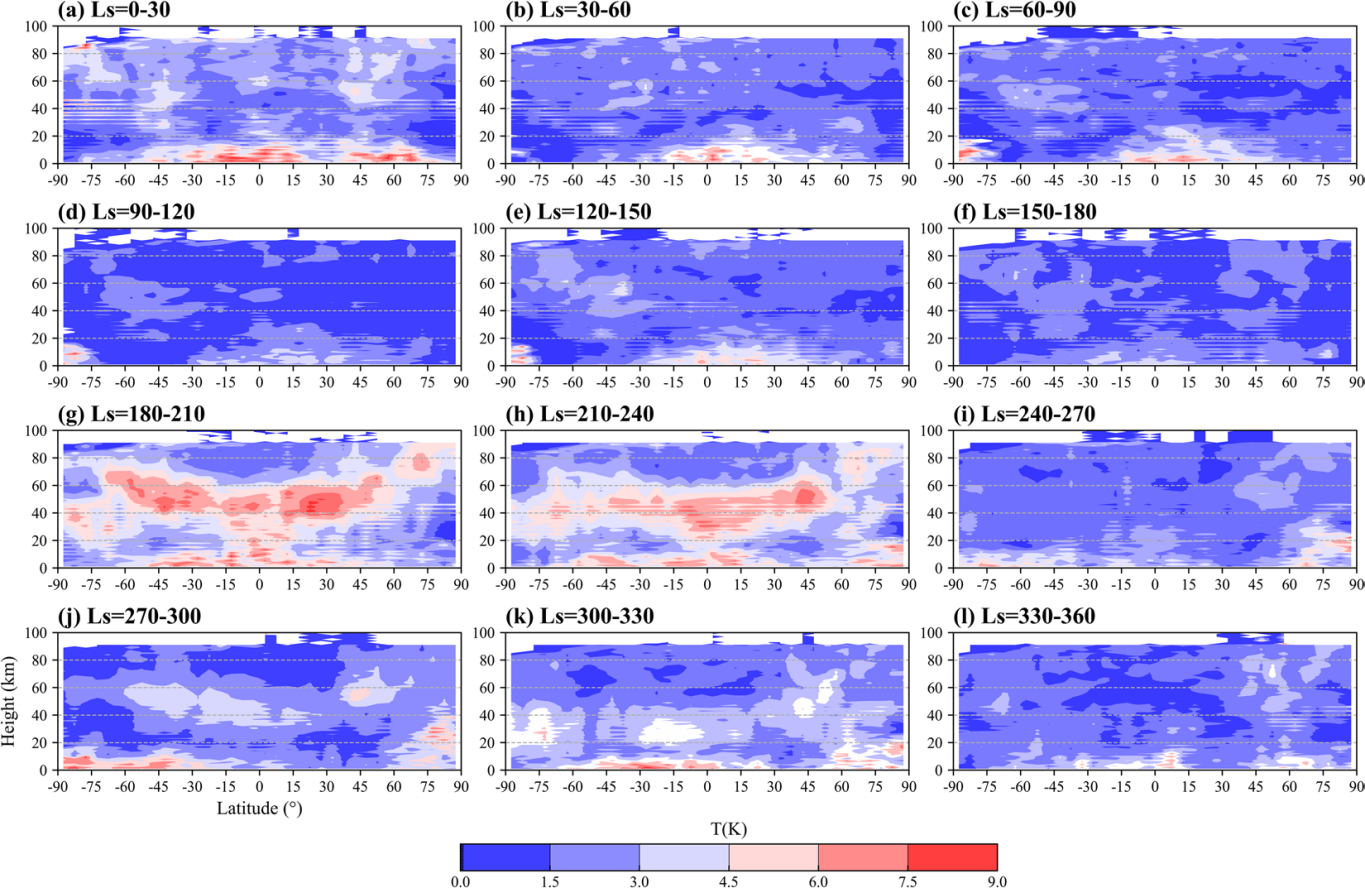
Height-latitude variations of DW1 temperature amplitude in K.

**Fig. 5:**
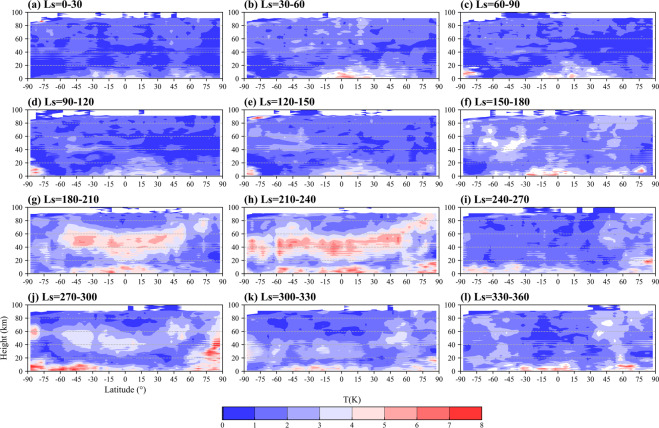
Same as Fig. 4 but for DE1 amplitude in K.

The temporal and spatial characteristics of Martian atmospheric tides are also maintained well. In Fig. 4, 5, the high-value area of DW1 and DE1 amplitude at nearly 50 km (or nearly 1 Pa) and near-surface due to the heat generated by solar absorption of dust particles^93^ during dust seasons are represented (Figs. 4g,h, 5g,h). The Doppler shift effects of zonal wind on the tides sustained by the dataset can be seen in the opposite hemisphere of DW1 and DE1 since the tide with opposite propagating direction to the zonal wind will be Doppler-shifted to higher frequencies and become less susceptible to dissipation^94^. The tropospheric westerly (easterly) in the mid-latitudes of the northern (southern) hemisphere induces the relatively strong DW1 (DE1) there near the tropopause, which is readily evident during the dust storm^72,95^ as shown in Figs. 4g,h, 5g,h.

### Performance of representing wave signatures at different altitudes

**Fig. 6:**
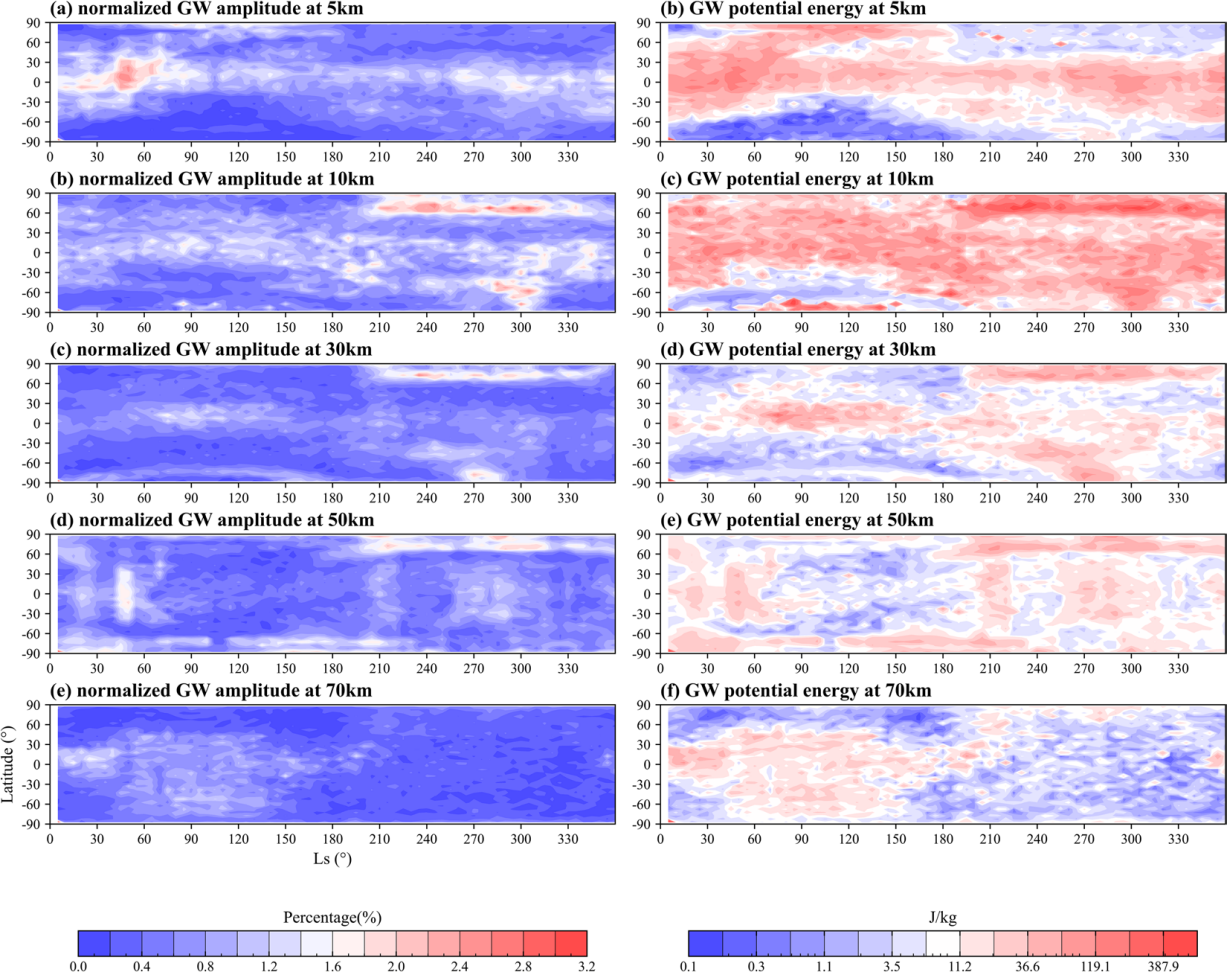
Zonal mean climatology of Martian atmospheric GW amplitude normalized by background temperature (left) and potential energy (right) within the whole MY at 5 km (**a, f**), 10 km (**b, g**), 30 km (**c, h**), 50 km (**d, i**) and 70 km (**e, j**) altitude.

The climatology GWs at five altitudes (three in the troposphere: 5, 10, 30 km and two in mesosphere: 50, 70 km) covering the whole Martian year show the GWs perturbations and potential energy with similar zonal distribution (Fig. 6). In the first half of the year (Ls = 0~180°), the maximum value of GWs perturbations in the lower atmosphere below 30 km altitude appears in the equatorial tropic region, which may be related to the mountainous topography in this area. This result is consistent with the conclusion of Creasey^10^. Above 30 km, the large GWs perturbations extend to the middle to high latitudes of the southern hemisphere and even to the polar regions. The most significant feature is the appearance of a large value belt in the northern polar region in the second half of the year (Ls = 180°~360°), but it is not obvious at 70 km. Throughout the year, GWs activity is more active in autumn and winter than in spring and summer. From an altitude perspective, the GWs in the lower layers of Mars is more active than that in the upper atmosphere in the range of 5–70 km.

**Fig. 7:**
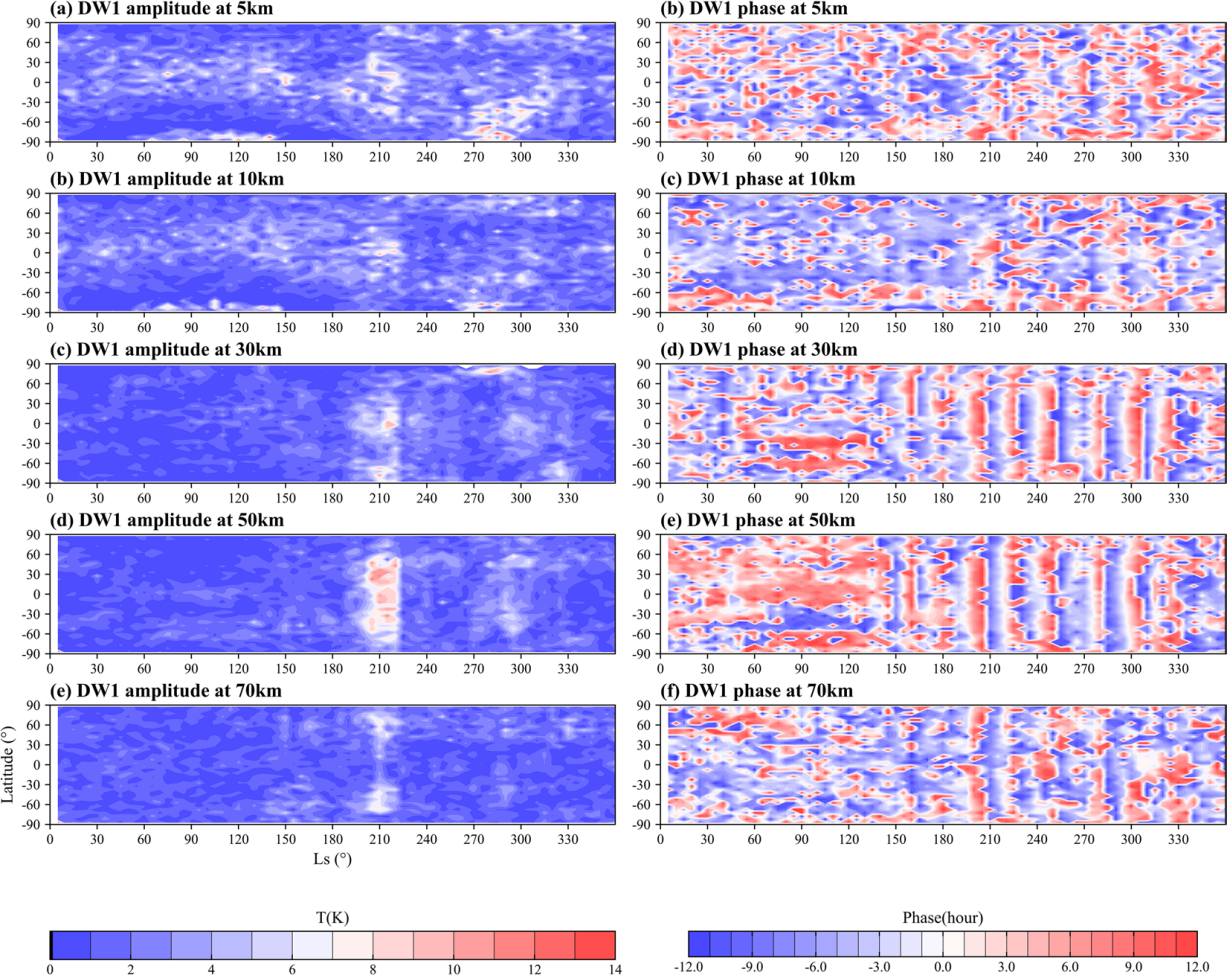
Zonal mean climatology of Martian atmospheric DW1 amplitude (left) and phase (right) within the whole MY at 5 km (**a, f**), 10 km (**b, g**), 30 km (**c, h**), 50 km (**d, i**) and 70 km (**e, j**) altitude.

As shown in Fig. 7c–e, the DW1 amplitudes from 30 km to 70 km are enhanced obviously near 210° Ls due to the dust storm. If we look at the changes at each altitude on the 210° Ls axis, we see that the tides first increase with altitude and then weaken with altitude after 50 km in the dust storm background, but are still significantly stronger than in the absence of a dust storm background. The dense staggered vertical bars of positive and negative values after 180° Ls were caused by the enhancement of upward propagation of thermal tides in the dusty season, which is more obvious at 30 and 50 km altitude (Fig. 7h,i) corresponding to the evident strong tides in Fig. 7c,d.

### Observational filter effects

The instrument visibility and observation geometry would influence the parts of the gravity wave spectrum observed^96,97^, leading to the observational filter effects of the dataset. Mixed gravity wave spectrum would be sampled by mixing perturbations from background temperature taken from a mixture of limb, nadir, occultation and entry accelerometer retrievals. Indeed, it is likely that different parts of the spectrum would be mixed in different ratios, resulting in a highly heterogeneous gravity wave results of the dataset. Despite all this, the dataset has to focus on the general composite gravity wave instead of the frequency dependent gravity wave due to the severe incompleteness of data coverage of single spacecraft. A single spacecraft could provide data with coverage on the order from ten percent to one thousandth, which is too small to retain the gravity wave of its specific spectrum and thus it is difficult to gain the gravity wave in the frequency domain. In addition, it was found that the observation filtering effects would not affect data reflection of the real gravity wave state in the atmosphere^96^. Consequently, the gravity wave calculated in the dataset represents the general composite gravity wave rather than gravity wave in the frequency domain.

## Supplementary information

Supplementary - Brief Data Descriptor
