# Supplementary material for: Observation based climatology Martian atmospheric waves perturbation Datasets
Source: Sci Data. 2023 Jan 3;10:4. doi: 10.1038/s41597-022-01909-y (PMC9810594; doi:10.1038/s41597-022-01909-y)
Supplement: Supplementary file 1 — Supplementary - Brief Data Descriptor [file 41597_2022_1909_MOESM1_ESM.pdf]

Brief Data Descriptor

The file contains a list of variables and a brief description of each.

Brief Data Descriptor

brief description of dataset 2
Variables 3

brief description of dataset

**Data type**	Dimension	Temperature	Gravity-wave	tide
**Variables**	Ls<br>Lt<br>Altitude<br>Latitude<br>Longitude	T (temperature)<br>T_sd (standard deviation)<br>T_I (instrument number)<br>T_D (data point number)	gravity waves'<br><br>E (potential enerygy)<br>pc_dt (amplitude)	amplitude and phase of<br><br>DW1, DW2, DW3, DW4, DW5,<br>DE1, DE2, DE3, DE4, DE5,<br>SW1, SW2, SW3, SW4, SW5,<br>SE1, SE2, SE3, SE4, SE5,<br>SPW1, SPW2, SPW3, SPW4, SPW5,<br>DS0, SS0, T_bg
**Dimension**	Ls (72)<br>Lt (12)<br>Altitude (100)<br>Latitude (36)<br>Longitude (36)	Ls (72)<br>Lt (12)<br>Altitude (100)<br>Latitude (36)<br>Longitude (36)	Ls (72)<br>Lt (12)<br>Altitude (100)<br>Latitude (36)<br>Longitude (36)	Ls (72)<br>Altitude (100)<br>Latitude (36)
**Level**		Level 1	Level 2	Level 2

General description of data is shown on the table above.

The 'MAWPD_XX.nc' file contains three kind of variables including 'dimension variables', 'Five dimensional variables', and 'Three dimensional variables'. Specifically, dimension variables 'Solar_Longitude', 'Local_Time',' Altitude', 'Latitude', and 'Longitude' are five dimensions called Ls, Lt, Altitude, Latitude, Longitude, respectively.

Five dimensional variables 'Temperature', 'Temperature_sd', 'Temperature_I', 'Temperature_D', 'GW', and 'E' are the air temperature (K), standard deviation of temperature (K), instrument number, data point number, gravity wave amplitude normalized by background temperature (%), and potential energy of gravity wave (J*kg-1) in the entire covered spatio-temporal domain, respectively. They are storage in 'MAWPD_v2.0_T.nc', 'MAWPD_v2.0_T_sd.nc', 'MAWPD_v2.0_T_I.nc', 'MAWPD_v2.0_T_D.nc', 'MAWPD_v2.0_GW_NA.nc', and 'MAWPD_v2.0_GW_PE.nc', respectively.

Five-dimensional variable 'Temperature' represents the climatology atmospheric state in the entire domain completely and sustain the calculation for 'GW', and 'E'. The super observation standard deviation 'Temperature_sd' of air temperature in the grid is a proxy of observation error, while the number of instruments contributing data to the grid 'Temperature_I' and number of data points in the grid 'Temperature_D' tell users how heavily it is constrained by data. Five-dimensional variable 'GW', and 'E' represents the climatology gravity waves in the entire domain.

Three dimensional variables 'amp_T_XXX' and 'phs_T_XXX' are the daily and zonally mean amplitude and phase of the specific tidal wave. For example, 'amp_T_DW1' and 'phs_T_DW1' are the daily and zonally amplitude and phase of the migrating diurnal tide (DW1). They are all storaged in 'MAWPD_v2.0_tide.nc'.

Variables

MAWPD_v2.0_T.nc

Format:

netcdf4_classic

Dimensions:

Solar_Longitude = 72

Local_Time = 12

Altitude = 100

Latitude = 36

Longitude = 36

Variables:

Solar_Longitude

Size: 72x1

Dimensions: Solar_Longitude

Datatype: double

Attributes:

units = 'degree'

creation_date = '28-Aug-2022 08:52:18'

description = 'solar longitude or Ls is the ecliptic longitude of the sun, i.e. the position of the sun on the celestial sphere along the ecliptic'

Local_Time

Size: 12x1

Dimensions: Local_Time

Datatype: double

Attributes:

units = 'hour'

creation_date = '28-Aug-2022 08:52:18'

description = 'time at a particular place as measured from the sun transit over the meridian at that place is defined as noon'

Altitude

Size: 100x1

Dimensions: Altitude

Datatype: double

Attributes:

units = 'km'

creation_date = '28-Aug-2022 08:52:18'

description = 'distance from the ground'

Latitude

Size: 36x1

Dimensions: Latitude

Datatype: double
Attributes:
units = 'degree'
creation_date = '28-Aug-2022 08:52:18'
description = 'latitude from -90 degree to 90 degree '

Longitude
Size: 36x1
Dimensions: Longitude
Datatype: double
Attributes:
units = 'degree'
creation_date = '28-Aug-2022 08:52:18'
description = 'longitude from -180 degree to 180 degree
'

Temperature
Size: 72x12x100x36x36
Dimensions: Solar_Longitude,Local_Time,Altitude,Latitude,Longitude
Datatype: double
Attributes:
units = 'K'
creation_date = '28-Aug-2022 08:52:18'
description = 'air temperature'

MAWPD_v2.0_T_sd.nc

Format:
netcdf4_classic
Dimensions:
Solar_Longitude = 72
Local_Time = 12
Altitude = 100
Latitude = 36
Longitude = 36
Variables:
Solar_Longitude
Size: 72x1
Dimensions: Solar_Longitude
Datatype: double
Attributes:
units = 'degree'
creation_date = '06-Nov-2022 18:00:20'
description = 'solar longitude or Ls is the ecliptic longitude of the sun, i.e. the position of the sun on the celestial sphere along the ecliptic'

Local_Time
Size: 12x1
Dimensions: Local_Time
Datatype: double
Attributes:
units = 'hour'
creation_date = '06-Nov-2022 18:00:20'
description = 'time at a particular place as measured from the sun transit over the meridian at that place is defined as noon'

Altitude
Size: 100x1
Dimensions: Altitude
Datatype: double
Attributes:
units = 'km'
creation_date = '06-Nov-2022 18:00:20'
description = 'distance from the ground'

Latitude
Size: 36x1
Dimensions: Latitude
Datatype: double
Attributes:
units = 'degree'
creation_date = '06-Nov-2022 18:00:20'
description = 'latitude from -90 degree to 90 degree '

Longitude
Size: 36x1
Dimensions: Longitude
Datatype: double
Attributes:
units = 'degree'
creation_date = '06-Nov-2022 18:00:20'
description = 'longitude from -180 degree to 180 degree '

Temperature_sd
Size: 72x12x100x36x36
Dimensions: Solar_Longitude,Local_Time,Altitude,Latitude,Longitude
Datatype: double
Attributes:
units = 'K'
creation_date = '06-Nov-2022 18:00:20'
description = 'super observation standard deviation of air temperature in the grid'

Format:
netcdf4_classic
Dimensions:
Solar_Longitude = 72
Local_Time = 12
Altitude = 100
Latitude = 36
Longitude = 36
Variables:
Solar_Longitude
Size: 72x1
Dimensions: Solar_Longitude
Datatype: double
Attributes:
units = 'degree'
creation_date = '06-Nov-2022 09:12:58'
description = 'solar longitude or Ls is the ecliptic longitude of the sun, i.e. the position of the sun on the celestial sphere along the ecliptic'
Local_Time
Size: 12x1
Dimensions: Local_Time
Datatype: double
Attributes:
units = 'hour'
creation_date = '06-Nov-2022 09:12:58'
description = 'time at a particular place as measured from the sun transit over the meridian at that place is defined as noon'
Altitude
Size: 100x1
Dimensions: Altitude
Datatype: double
Attributes:
units = 'km'
creation_date = '06-Nov-2022 09:12:58'
description = 'distance from the ground'
Latitude
Size: 36x1
Dimensions: Latitude
Datatype: double
Attributes:
units = 'degree'

creation_date = '06-Nov-2022 09:12:58'
description = 'latitude from -90 degree to 90 degree '

Longitude

Size: 36x1

Dimensions: Longitude

Datatype: double

Attributes:

units = 'degree'
creation_date = '06-Nov-2022 09:12:58'
description = 'longitude from -180 degree to 180 degree '

Temperature_I

Size: 72x12x100x36x36

Dimensions: Solar_Longitude,Local_Time,Altitude,Latitude,Longitude

Datatype: double

Attributes:

units = 'dimensionless'
creation_date = '06-Nov-2022 09:12:58'
description = 'number of instruments contributing data to the grid'

MAWPD_v2.0_T_D.nc

Format:

netcdf4_classic

Dimensions:

Solar_Longitude = 72
Local_Time = 12
Altitude = 100
Latitude = 36
Longitude = 36

Variables:

Solar_Longitude

Size: 72x1

Dimensions: Solar_Longitude

Datatype: double

Attributes:

units = 'degree'
creation_date = '06-Nov-2022 09:13:03'
description = 'solar longitude or Ls is the ecliptic longitude of the sun, i.e. the position of the sun on the celestial sphere along the ecliptic'

Local_Time

Size: 12x1

Dimensions: Local_Time
Datatype: double
Attributes:
units = 'hour'
creation_date = '06-Nov-2022 09:13:03'
description = 'time at a particular place as measured from the sun transit over the meridian at that place is defined as noon'

Altitude
Size: 100x1
Dimensions: Altitude
Datatype: double
Attributes:
units = 'km'
creation_date = '06-Nov-2022 09:13:03'
description = 'distance from the ground'

Latitude
Size: 36x1
Dimensions: Latitude
Datatype: double
Attributes:
units = 'degree'
creation_date = '06-Nov-2022 09:13:03'
description = 'latitude from -90 degree to 90 degree '

Longitude
Size: 36x1
Dimensions: Longitude
Datatype: double
Attributes:
units = 'degree'
creation_date = '06-Nov-2022 09:13:03'
description = 'longitude from -180 degree to 180 degree '

Temperature_D
Size: 72x12x100x36x36
Dimensions: Solar_Longitude,Local_Time,Altitude,Latitude,Longitude
Datatype: double
Attributes:
units = 'dimensionless'
creation_date = '06-Nov-2022 09:13:03'
description = 'Number of data points in the grid'

MAWPD_v2.0_GW_NA.nc

Format:

netcdf4_classic

Dimensions:

Solar_Longitude = 72

Local_Time = 12

Altitude = 100

Latitude = 36

Longitude = 36

Variables:

Solar_Longitude

Size: 72x1

Dimensions: Solar_Longitude

Datatype: double

Attributes:

units = 'degree'

creation_date = '28-Aug-2022 08:52:22'

description = 'solar longitude or Ls is the ecliptic longitude of the sun, i.e. the position of the sun on the celestial sphere along the ecliptic'

Local_Time

Size: 12x1

Dimensions: Local_Time

Datatype: double

Attributes:

units = 'hour'

creation_date = '28-Aug-2022 08:52:22'

description = 'time at a particular place as measured from the sun transit over the meridian at that place is defined as noon'

Altitude

Size: 100x1

Dimensions: Altitude

Datatype: double

Attributes:

units = 'km'

creation_date = '28-Aug-2022 08:52:22'

description = 'distance from the ground'

Latitude

Size: 36x1

Dimensions: Latitude

Datatype: double

Attributes:

units = 'degree'

creation_date = '28-Aug-2022 08:52:22'

description = 'latitude from -90 degree to 90 degree '

Longitude

Size: 36x1
Dimensions: Longitude
Datatype: double
Attributes:
units = 'degree'
creation_date = '28-Aug-2022 08:52:22'
description = 'longitude from -180 degree to 180 degree
'
GW
Size: 72x12x100x36x36
Dimensions: Solar_Longitude,Local_Time,Altitude,Latitude,Longitude
Datatype: double
Attributes:
units = 'Percent(%)'
creation_date = '28-Aug-2022 08:52:22'
description = 'gravity wave amplitude normalized by background temperature'

MAWPD_v2.0_GW_PE.nc

Format:
netcdf4_classic
Dimensions:
Solar_Longitude = 72
Local_Time = 12
Altitude = 100
Latitude = 36
Longitude = 36
Variables:
Solar_Longitude
Size: 72x1
Dimensions: Solar_Longitude
Datatype: double
Attributes:
units = 'degree'
creation_date = '28-Aug-2022 08:52:27'
description = 'solar longitude or Ls is the ecliptic longitude of the sun, i.e. the position of the sun on the celestial sphere along the ecliptic'
Local_Time
Size: 12x1
Dimensions: Local_Time
Datatype: double
Attributes:

units = 'hour'
creation_date = '28-Aug-2022 08:52:27'
description = 'time at a particular place as measured from the sun transit over the meridian at that place is defined as noon'

Altitude
Size: 100x1
Dimensions: Altitude
Datatype: double
Attributes:
units = 'km'
creation_date = '28-Aug-2022 08:52:27'
description = 'distance from the ground'

Latitude
Size: 36x1
Dimensions: Latitude
Datatype: double
Attributes:
units = 'degree'
creation_date = '28-Aug-2022 08:52:27'
description = 'latitude from -90 degree to 90 degree '

Longitude
Size: 36x1
Dimensions: Longitude
Datatype: double
Attributes:
units = 'degree'
creation_date = '28-Aug-2022 08:52:27'
description = 'longitude from -180 degree to 180 degree '

E
Size: 72x12x100x36x36
Dimensions: Solar_Longitude,Local_Time,Altitude,Latitude,Longitude
Datatype: double
Attributes:
units = 'J*kg-1'
creation_date = '28-Aug-2022 08:52:27'
description = 'gravity wave potential energy'

MAWPD_v2.0_tide.nc

Format:
netcdf4_classic
Dimensions:

Solar_Longitude = 72
Local_Time = 12
Altitude = 100
Latitude = 36
Longitude = 36

Variables:

Solar_Longitude

Size: 72x1
Dimensions: Solar_Longitude
Datatype: double
Attributes:

units = 'degree'
creation_date = '06-Nov-2022 09:03:59'
description = 'solar longitude or Ls is the ecliptic longitude of the sun, i.e. the position of the sun on the celestial sphere along the ecliptic'

Local_Time

Size: 12x1
Dimensions: Local_Time
Datatype: double
Attributes:

units = 'hour'
creation_date = '06-Nov-2022 09:03:59'
description = 'time at a particular place as measured from the sun transit over the meridian at that place is defined as noon'

Altitude

Size: 100x1
Dimensions: Altitude
Datatype: double
Attributes:

units = 'km'
creation_date = '06-Nov-2022 09:03:59'
description = 'distance from the ground'

Latitude

Size: 36x1
Dimensions: Latitude
Datatype: double
Attributes:

units = 'degree'
creation_date = '06-Nov-2022 09:03:59'
description = 'latitude from -90 degree to 90 degree '

Longitude

Size: 36x1
Dimensions: Longitude
Datatype: double

Attributes:
units = 'degree'
creation_date = '06-Nov-2022 09:03:59'
description = 'longitude from -180 degree to 180 degree
'
amp_T_DE1
Size: 72x100x36
Dimensions: Solar_Longitude,Altitude,Latitude
Datatype: double
Attributes:
units = 'K'
creation_date = '06-Nov-2022 09:03:59'
description = 'amplitude of diurnal eastward propagating tide with the zonal wavenumber of 1'
amp_T_DE2
Size: 72x100x36
Dimensions: Solar_Longitude,Altitude,Latitude
Datatype: double
Attributes:
units = 'K'
creation_date = '06-Nov-2022 09:03:59'
description = 'amplitude of diurnal eastward propagating tide with the zonal wavenumber of 2'
amp_T_DE3
Size: 72x100x36
Dimensions: Solar_Longitude,Altitude,Latitude
Datatype: double
Attributes:
units = 'K'
creation_date = '06-Nov-2022 09:03:59'
description = 'amplitude of diurnal eastward propagating tide with the zonal wavenumber of 3'
amp_T_DE4
Size: 72x100x36
Dimensions: Solar_Longitude,Altitude,Latitude
Datatype: double
Attributes:
units = 'K'
creation_date = '06-Nov-2022 09:03:59'
description = 'amplitude of diurnal eastward propagating tide with the zonal wavenumber of 4'
amp_T_DE5
Size: 72x100x36
Dimensions: Solar_Longitude,Altitude,Latitude

Datatype: double
Attributes:
units = 'K'
creation_date = '06-Nov-2022 09:03:59'
description = 'amplitude of diurnal eastward propagating tide with the zonal wavenumber of 5'

amp_T_DS0
Size: 72x100x36
Dimensions: Solar_Longitude,Altitude,Latitude
Datatype: double
Attributes:
units = 'K'
creation_date = '06-Nov-2022 09:03:59'
description = 'amplitude of diurnal zonally symmetric tide'

amp_T_DW1
Size: 72x100x36
Dimensions: Solar_Longitude,Altitude,Latitude
Datatype: double
Attributes:
units = 'K'
creation_date = '06-Nov-2022 09:03:59'
description = 'amplitude of diurnal westward propagating tide with the zonal wavenumber of 1'

amp_T_DW2
Size: 72x100x36
Dimensions: Solar_Longitude,Altitude,Latitude
Datatype: double
Attributes:
units = 'K'
creation_date = '06-Nov-2022 09:03:59'
description = 'amplitude of diurnal westward propagating tide with the zonal wavenumber of 2'

amp_T_DW3
Size: 72x100x36
Dimensions: Solar_Longitude,Altitude,Latitude
Datatype: double
Attributes:
units = 'K'
creation_date = '06-Nov-2022 09:03:59'
description = 'amplitude of diurnal westward propagating tide with the zonal wavenumber of 3'

amp_T_DW4
Size: 72x100x36
Dimensions: Solar_Longitude,Altitude,Latitude

Datatype: double
Attributes:
units = 'K'
creation_date = '06-Nov-2022 09:03:59'
description = 'amplitude of diurnal westward propagating tide with the zonal wavenumber of 4'
amp_T_DW5
Size: 72x100x36
Dimensions: Solar_Longitude,Altitude,Latitude
Datatype: double
Attributes:
units = 'K'
creation_date = '06-Nov-2022 09:03:59'
description = 'amplitude of diurnal westward propagating tide with the zonal wavenumber of 5'
amp_T_SE1
Size: 72x100x36
Dimensions: Solar_Longitude,Altitude,Latitude
Datatype: double
Attributes:
units = 'K'
creation_date = '06-Nov-2022 09:03:59'
description = 'amplitude of semi-diurnal eastward propagating tide with the zonal wavenumber of 1'
amp_T_SE2
Size: 72x100x36
Dimensions: Solar_Longitude,Altitude,Latitude
Datatype: double
Attributes:
units = 'K'
creation_date = '06-Nov-2022 09:03:59'
description = 'amplitude of semi-diurnal eastward propagating tide with the zonal wavenumber of 2'
amp_T_SE3
Size: 72x100x36
Dimensions: Solar_Longitude,Altitude,Latitude
Datatype: double
Attributes:
units = 'K'
creation_date = '06-Nov-2022 09:03:59'
description = 'amplitude of semi-diurnal eastward propagating tide with the zonal wavenumber of 3'
amp_T_SE4
Size: 72x100x36

Dimensions: Solar_Longitude,Altitude,Latitude
Datatype: double
Attributes:
units = 'K'
creation_date = '06-Nov-2022 09:03:59'
description = 'amplitude of semi-diurnal eastward propagating tide with the zonal wavenumber of 4'

amp_T_SE5
Size: 72x100x36
Dimensions: Solar_Longitude,Altitude,Latitude
Datatype: double
Attributes:
units = 'K'
creation_date = '06-Nov-2022 09:03:59'
description = 'amplitude of semi-diurnal eastward propagating tide with the zonal wavenumber of 5 '

amp_T_SPW1
Size: 72x100x36
Dimensions: Solar_Longitude,Altitude,Latitude
Datatype: double
Attributes:
units = 'K'
creation_date = '06-Nov-2022 09:03:59'
description = 'amplitude of stationary planetary wave with the zonal wavenumber of 1'

amp_T_SPW2
Size: 72x100x36
Dimensions: Solar_Longitude,Altitude,Latitude
Datatype: double
Attributes:
units = 'K'
creation_date = '06-Nov-2022 09:03:59'
description = 'amplitude of stationary planetary wave with the zonal wavenumber of 2'

amp_T_SPW3
Size: 72x100x36
Dimensions: Solar_Longitude,Altitude,Latitude
Datatype: double
Attributes:
units = 'K'
creation_date = '06-Nov-2022 09:03:59'
description = 'amplitude of stationary planetary wave with the zonal wavenumber of 3'

amp_T_SPW4

Size: 72x100x36
Dimensions: Solar_Longitude,Altitude,Latitude
Datatype: double
Attributes:
units = 'K'
creation_date = '06-Nov-2022 09:03:59'
description = 'amplitude of stationary planetary wave with the zonal wavenumber of 4'

amp_T_SPW5
Size: 72x100x36
Dimensions: Solar_Longitude,Altitude,Latitude
Datatype: double
Attributes:
units = 'K'
creation_date = '06-Nov-2022 09:03:59'
description = 'amplitude of stationary planetary wave with the zonal wavenumber of 5 '

amp_T_SS0
Size: 72x100x36
Dimensions: Solar_Longitude,Altitude,Latitude
Datatype: double
Attributes:
units = 'K'
creation_date = '06-Nov-2022 09:03:59'
description = 'amplitude of semi-diurnal zonally symmetric tide'

amp_T_SW1
Size: 72x100x36
Dimensions: Solar_Longitude,Altitude,Latitude
Datatype: double
Attributes:
units = 'K'
creation_date = '06-Nov-2022 09:03:59'
description = 'amplitude of semi-diurnal westward propagating tide with the zonal wavenumber of 1'

amp_T_SW2
Size: 72x100x36
Dimensions: Solar_Longitude,Altitude,Latitude
Datatype: double
Attributes:
units = 'K'
creation_date = '06-Nov-2022 09:03:59'
description = 'amplitude of semi-diurnal westward propagating tide with the zonal wavenumber of 2'

amp_T_SW3

Size: 72x100x36

Dimensions: Solar_Longitude,Altitude,Latitude

Datatype: double

Attributes:

units = 'K'

creation_date = '06-Nov-2022 09:03:59'

description = 'amplitude of semi-diurnal westward propagating tide with the zonal wavenumber of 3'

amp_T_SW4

Size: 72x100x36

Dimensions: Solar_Longitude,Altitude,Latitude

Datatype: double

Attributes:

units = 'K'

creation_date = '06-Nov-2022 09:03:59'

description = 'amplitude of semi-diurnal westward propagating tide with the zonal wavenumber of 4'

amp_T_SW5

Size: 72x100x36

Dimensions: Solar_Longitude,Altitude,Latitude

Datatype: double

Attributes:

units = 'K'

creation_date = '06-Nov-2022 09:03:59'

description = 'amplitude of semi-diurnal westward propagating tide with the zonal wavenumber of 5'

amp_T_bg

Size: 72x100x36

Dimensions: Solar_Longitude,Altitude,Latitude

Datatype: double

Attributes:

units = 'K'

creation_date = '06-Nov-2022 09:03:59'

description = 'amplitude of the background temperature'

phs_T_DE1

Size: 72x100x36

Dimensions: Solar_Longitude,Altitude,Latitude

Datatype: double

Attributes:

units = 'K'

creation_date = '06-Nov-2022 09:03:59'

description = 'phase of diurnal eastward propagating tide with the zonal wavenumber of 1'

phs_T_DE2
Size: 72x100x36
Dimensions: Solar_Longitude,Altitude,Latitude
Datatype: double
Attributes:
units = 'K'
creation_date = '06-Nov-2022 09:03:59'
description = 'phase of diurnal eastward propagating tide with the zonal wavenumber of 2'
phs_T_DE3
Size: 72x100x36
Dimensions: Solar_Longitude,Altitude,Latitude
Datatype: double
Attributes:
units = 'K'
creation_date = '06-Nov-2022 09:03:59'
description = 'phase of diurnal eastward propagating tide with the zonal wavenumber of 3'
phs_T_DE4
Size: 72x100x36
Dimensions: Solar_Longitude,Altitude,Latitude
Datatype: double
Attributes:
units = 'K'
creation_date = '06-Nov-2022 09:03:59'
description = 'phase of diurnal eastward propagating tide with the zonal wavenumber of 4'
phs_T_DE5
Size: 72x100x36
Dimensions: Solar_Longitude,Altitude,Latitude
Datatype: double
Attributes:
units = 'K'
creation_date = '06-Nov-2022 09:03:59'
description = 'phase of diurnal eastward propagating tide with the zonal wavenumber of 5'
phs_T_DS0
Size: 72x100x36
Dimensions: Solar_Longitude,Altitude,Latitude
Datatype: double
Attributes:
units = 'K'
creation_date = '06-Nov-2022 09:03:59'
description = 'phase of diurnal zonally symmetric tide'

phs_T_DW1
Size: 72x100x36
Dimensions: Solar_Longitude,Altitude,Latitude
Datatype: double
Attributes:
units = 'K'
creation_date = '06-Nov-2022 09:03:59'
description = 'phase of diurnal westward propagating tide with the zonal wavenumber of 1'

phs_T_DW2
Size: 72x100x36
Dimensions: Solar_Longitude,Altitude,Latitude
Datatype: double
Attributes:
units = 'K'
creation_date = '06-Nov-2022 09:03:59'
description = 'phase of diurnal westward propagating tide with the zonal wavenumber of 2'

phs_T_DW3
Size: 72x100x36
Dimensions: Solar_Longitude,Altitude,Latitude
Datatype: double
Attributes:
units = 'K'
creation_date = '06-Nov-2022 09:03:59'
description = 'phase of diurnal westward propagating tide with the zonal wavenumber of 3'

phs_T_DW4
Size: 72x100x36
Dimensions: Solar_Longitude,Altitude,Latitude
Datatype: double
Attributes:
units = 'K'
creation_date = '06-Nov-2022 09:03:59'
description = 'phase of diurnal westward propagating tide with the zonal wavenumber of 4'

phs_T_DW5
Size: 72x100x36
Dimensions: Solar_Longitude,Altitude,Latitude
Datatype: double
Attributes:
units = 'K'
creation_date = '06-Nov-2022 09:03:59'
description = 'phase of diurnal westward propagating tide with

the zonal wavenumber of 5'

phs_T_SE1

Size: 72x100x36

Dimensions: Solar_Longitude,Altitude,Latitude

Datatype: double

Attributes:

units = 'K'

creation_date = '06-Nov-2022 09:03:59'

description = 'phase of semi-diurnal eastward propagating tide with the zonal wavenumber of 1'

phs_T_SE2

Size: 72x100x36

Dimensions: Solar_Longitude,Altitude,Latitude

Datatype: double

Attributes:

units = 'K'

creation_date = '06-Nov-2022 09:03:59'

description = 'phase of semi-diurnal eastward propagating tide with the zonal wavenumber of 2'

phs_T_SE3

Size: 72x100x36

Dimensions: Solar_Longitude,Altitude,Latitude

Datatype: double

Attributes:

units = 'K'

creation_date = '06-Nov-2022 09:03:59'

description = 'phase of semi-diurnal eastward propagating tide with the zonal wavenumber of 3'

phs_T_SE4

Size: 72x100x36

Dimensions: Solar_Longitude,Altitude,Latitude

Datatype: double

Attributes:

units = 'K'

creation_date = '06-Nov-2022 09:03:59'

description = 'phase of semi-diurnal eastward propagating tide with the zonal wavenumber of 4'

phs_T_SE5

Size: 72x100x36

Dimensions: Solar_Longitude,Altitude,Latitude

Datatype: double

Attributes:

units = 'K'

creation_date = '06-Nov-2022 09:03:59'

description = 'phase of semi-diurnal eastward propagating tide with the zonal wavenumber of 5 '

phs_T_SPW1

Size: 72x100x36

Dimensions: Solar_Longitude,Altitude,Latitude

Datatype: double

Attributes:

units = 'K'

creation_date = '06-Nov-2022 09:03:59'

description = 'phase of stationary planetary wave with the zonal wavenumber of 1'

phs_T_SPW2

Size: 72x100x36

Dimensions: Solar_Longitude,Altitude,Latitude

Datatype: double

Attributes:

units = 'K'

creation_date = '06-Nov-2022 09:03:59'

description = 'phase of stationary planetary wave with the zonal wavenumber of 2'

phs_T_SPW3

Size: 72x100x36

Dimensions: Solar_Longitude,Altitude,Latitude

Datatype: double

Attributes:

units = 'K'

creation_date = '06-Nov-2022 09:03:59'

description = 'phase of stationary planetary wave with the zonal wavenumber of 3'

phs_T_SPW4

Size: 72x100x36

Dimensions: Solar_Longitude,Altitude,Latitude

Datatype: double

Attributes:

units = 'K'

creation_date = '06-Nov-2022 09:03:59'

description = 'phase of stationary planetary wave with the zonal wavenumber of 4'

phs_T_SPW5

Size: 72x100x36

Dimensions: Solar_Longitude,Altitude,Latitude

Datatype: double

Attributes:

units = 'K'

creation_date = '06-Nov-2022 09:03:59'
description = 'phase of stationary planetary wave with the zonal wavenumber of 5 '

phs_T_SS0
Size: 72x100x36
Dimensions: Solar_Longitude,Altitude,Latitude
Datatype: double
Attributes:
units = 'K'
creation_date = '06-Nov-2022 09:03:59'
description = 'phase of semi-diurnal zonally symmetric tide'

phs_T_SW1
Size: 72x100x36
Dimensions: Solar_Longitude,Altitude,Latitude
Datatype: double
Attributes:
units = 'K'
creation_date = '06-Nov-2022 09:03:59'
description = 'phase of semi-diurnal westward propagating tide with the zonal wavenumber of 1'

phs_T_SW2
Size: 72x100x36
Dimensions: Solar_Longitude,Altitude,Latitude
Datatype: double
Attributes:
units = 'K'
creation_date = '06-Nov-2022 09:03:59'
description = 'phase of semi-diurnal westward propagating tide with the zonal wavenumber of 2'

phs_T_SW3
Size: 72x100x36
Dimensions: Solar_Longitude,Altitude,Latitude
Datatype: double
Attributes:
units = 'K'
creation_date = '06-Nov-2022 09:03:59'
description = 'phase of semi-diurnal westward propagating tide with the zonal wavenumber of 3'

phs_T_SW4
Size: 72x100x36
Dimensions: Solar_Longitude,Altitude,Latitude
Datatype: double
Attributes:
units = 'K'

creation_date = '06-Nov-2022 09:03:59'
description = 'phase of semi-diurnal westward propagating tide with the zonal wavenumber of 4'

phs_T_SW5

Size: 72x100x36

Dimensions: Solar_Longitude,Altitude,Latitude

Datatype: double

Attributes:

units = 'K'

creation_date = '06-Nov-2022 09:03:59'

description = 'phase of semi-diurnal westward propagating tide with the zonal wavenumber of 5'

phs_T_bg

Size: 72x100x36

Dimensions: Solar_Longitude,Altitude,Latitude

Datatype: double

Attributes:

units = 'K'

creation_date = '06-Nov-2022 09:03:59'

description = 'phase of the background temperature '
